# Mutation Analysis of 2009 Pandemic Influenza A(H1N1) Viruses Collected in Japan during the Peak Phase of the Pandemic

**DOI:** 10.1371/journal.pone.0018956

**Published:** 2011-04-29

**Authors:** Jean-Étienne Morlighem, Shintaro Aoki, Mami Kishima, Mitsue Hanami, Chihiro Ogawa, Amadu Jalloh, Yukari Takahashi, Yuki Kawai, Satomi Saga, Eiji Hayashi, Toshiaki Ban, Shinyu Izumi, Akira Wada, Masayuki Mano, Megumu Fukunaga, Yoshiyuki Kijima, Masashi Shiomi, Kaoru Inoue, Takeshi Hata, Yukihiro Koretsune, Koichiro Kudo, Yuji Himeno, Aizan Hirai, Kazuo Takahashi, Yuko Sakai-Tagawa, Kiyoko Iwatsuki-Horimoto, Yoshihiro Kawaoka, Yoshihide Hayashizaki, Toshihisa Ishikawa

**Affiliations:** 1 Omics Science Center, RIKEN Yokohama Institute, Yokohama, Japan; 2 Chiba Prefectural Togane Hospital, Togane, Japan; 3 Isumi Medical Center, Isumi, Japan; 4 National Center for Global Health and Medicine, Tokyo, Japan; 5 National Hospital Organization, Osaka National Hospital, Osaka, Japan; 6 Toyonaka Municipal Hospital, Toyonaka, Japan; 7 Higashi-Osaka City General Hospital, Higashi-Osaka, Japan; 8 Osaka City General Hospital, Osaka, Japan; 9 Osaka Prefectural Institute of Public Health, Osaka, Japan; 10 Institute of Medical Science, University of Tokyo, Tokyo, Japan; Johns Hopkins University - Bloomberg School of Public Health, United States of America

## Abstract

**Background:**

Pandemic influenza A(H1N1) virus infection quickly circulated worldwide in 2009. In Japan, the first case was reported in May 2009, one month after its outbreak in Mexico. Thereafter, A(H1N1) infection spread widely throughout the country. It is of great importance to profile and understand the situation regarding viral mutations and their circulation in Japan to accumulate a knowledge base and to prepare clinical response platforms before a second pandemic (pdm) wave emerges.

**Methodology:**

A total of 253 swab samples were collected from patients with influenza-like illness in the Osaka, Tokyo, and Chiba areas both in May 2009 and between October 2009 and January 2010. We analyzed partial sequences of the hemagglutinin (HA) and neuraminidase (NA) genes of the 2009 pdm influenza virus in the collected clinical samples. By phylogenetic analysis, we identified major variants of the 2009 pdm influenza virus and critical mutations associated with severe cases, including drug-resistance mutations.

**Results and Conclusions:**

Our sequence analysis has revealed that both HA-S220T and NA-N248D are major non-synonymous mutations that clearly discriminate the 2009 pdm influenza viruses identified in the very early phase (May 2009) from those found in the peak phase (October 2009 to January 2010) in Japan. By phylogenetic analysis, we found 14 micro-clades within the viruses collected during the peak phase. Among them, 12 were new micro-clades, while two were previously reported. Oseltamivir resistance-related mutations, i.e., NA-H275Y and NA-N295S, were also detected in sporadic cases in Osaka and Tokyo.

## Introduction

The 2009 pandemic (pdm) influenza A(H1N1) virus, a new strain of virus identified in Mexico in April 2009, spread quickly among humans worldwide [1]–[4] to cause the first influenza pandemic disease of the 21st century. As of April 2010, this swine-origin influenza virus had led to outbreaks on both local and global scales with severe consequences for human health and the global economy, resulting in about 18,000 deaths around the world [4].

On August 10, 2010, the WHO announced that the 2009 pdm A(H1N1) influenza had moved into the post-pandemic period [5]. In spite of this, however, localized outbreaks of various magnitudes continued. In fact, transmission of the 2009 pdm A(H1N1) influenza virus remained intense in certain parts of India and in the temperate southern hemisphere, particularly New Zealand and Australia [6]. There are concerns that this virus may mutate or reassort with other existing influenza viruses to give rise to more readily transmittable or more pathogenic viruses.

The 2009 pdm influenza virus is a triple combination comprising gene segments from both North American and Eurasian swine influenza and from avian influenza viruses. Namely, the 2009 pdm influenza A(H1N1) virus possesses PB2 and PA genes of North American avian virus origin, a PB1 gene of human H3N2 virus origin, HA (H1), NP, and NS genes of classical swine virus origin, and NA (N1) and M genes of Eurasian avian-like swine virus origin [7], [8]. In this regard, the 2009 pdm influenza A(H1N1) virus is unique. Unusual for influenza, the 2009 pdm influenza preferentially affected young adults and children, whereas elderly people generally showed preexisting immunity [9], [10]. The influenza virus envelope protein HA is a principal surface antigen. A comparison of amino acid sequences between the 2009 pdm A(H1N1) and previous influenza viruses revealed that the 2009 pdm A(H1N1) virus and the 1918 Spanish influenza viruses share some common signatures [11]. The 2D1 antibody from a survivor of the 1918 Spanish flu neutralized both 1918 and 2009 pdm H1N1 viruses, suggesting that the antibody's epitope is conserved in both pandemic viruses [12].

Since the outbreak of the 2009 pdm A(H1N1) influenza infection, a large-scale surveillance was carried out at the molecular level, and the evolutionary and spatial dynamics of the 2009 pdm A(H1N1) virus have been well characterized to date [13], [14]. The virus genome was found to have an extremely high evolutionary rate [15]. Phylogenetic analyses have shown that viruses in four major clusters have disseminated globally and co-circulated over time and space since April 2009 [13], [14].

In Japan, the first 2009 pdm A(H1N1) influenza case was reported on May 9, 2009, and that was followed by more than 200 reported cases in the Osaka and Kobe areas by May 21, 2009 [16]. Thereafter, the pandemic infection spread widely throughout Japan, where the numbers of influenza cases reported per sentinel provider peaked at 39.63 in November 2009, with over 200 fatal cases due to infection with the 2009 pdm influenza viruses. Shiino *et al.* analyzed the molecular evolution of the 2009 pdm A(H1N1) virus to find that a major part of the 75 strains isolated in Japan could be differentiated into 12 micro-clades [17]. Their analysis, however, was performed only for the samples collected from May until September, 2009, before the peak phase of the pandemic.

To gain insight into how the 2009 pdm H1N1 influenza virus evolved thereafter in Japan, we collected a total of 253 swab samples (influenza A-positive) both at the early beginning of infections in the Osaka area and in the peak phase (October 2009 to January 2010) of the pandemic stage in the Osaka, Tokyo, and Chiba areas. We isolated viral RNA from the collected samples and analyzed the sequences of genes encoding hemagglutinin (HA) and neuraminidase (NA). The phylogenic analysis data demonstrate that the 2009 pdm influenza viruses in Japan differed between the very early phase and the peak phase of the pandemic.

## Results

### Collection of samples and partial sequencing

**Figure 1:**
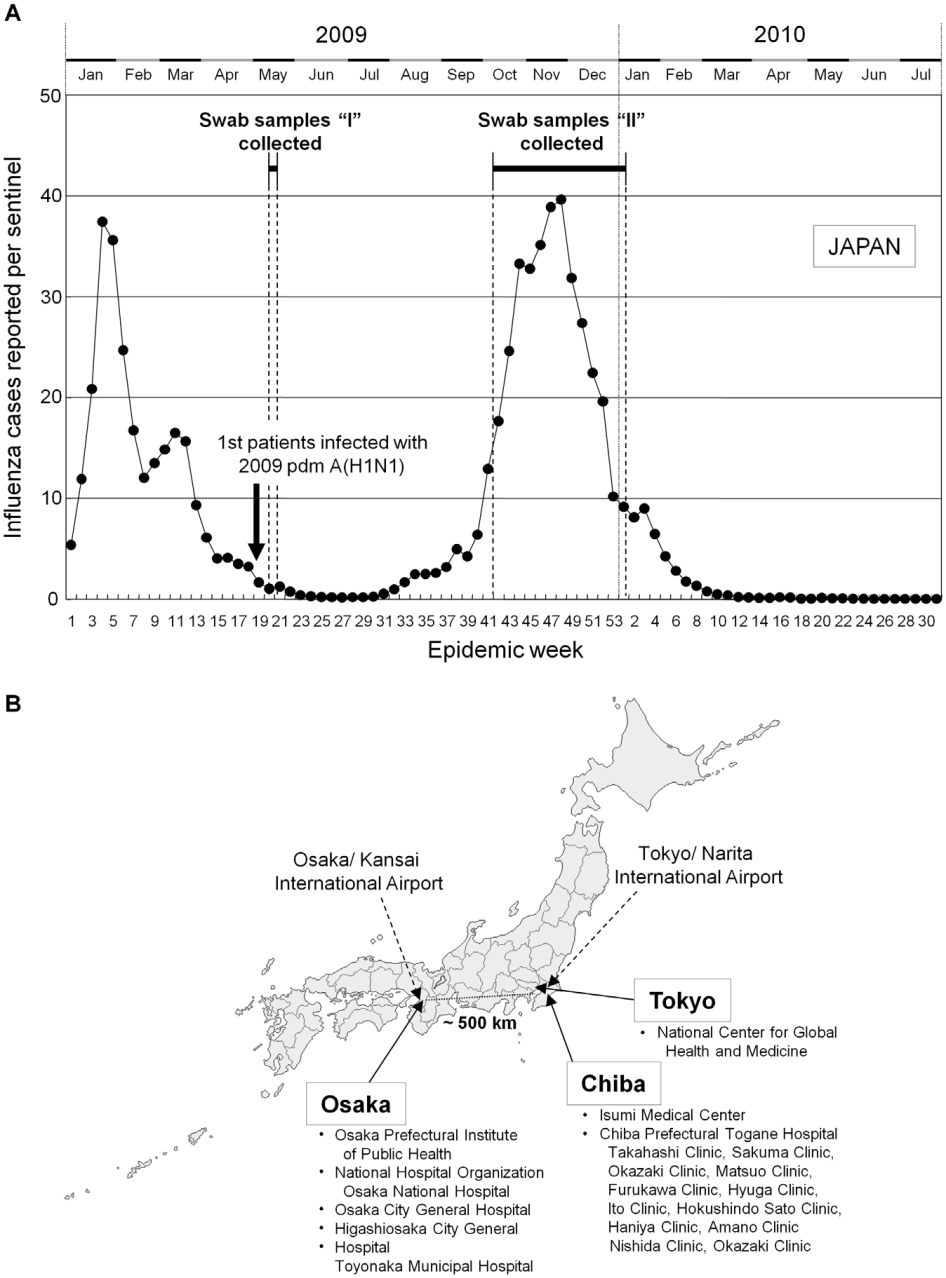
Collection of swab samples during initial and pandemic periods in Japan. (A) The number of influenza cases reported per sentinel provider in Japan and the collection of swab samples from outpatients in Osaka, Tokyo, and Chiba areas. Swab samples were collected during initial and pandemic periods that are indicated by “I” (May 16 to May 20, 2009) and “II” (October 13, 2009 to January 6, 2010), respectively. In Japan, the first patients (three Japanese men) infected with the 2009 pdm A(H1N1) were discovered on May 9 (week 19), 2009. Data on the number of influenza cases reported per sentinel provider in Japan are acquired from the pandemic influenza A(H1N1) situation report of the Infectious Disease Surveillance Center. The number of sentinel clinics and hospitals is dependent on the relative population of each public health center's jurisdiction and in consideration of enabling comprehension of the incidents within each entire prefecture. The influenza sentinel points thus comprise approximately 3,000 pediatric hospitals/clinics and 1,800 internist hospitals/clinics in Japan. (B) Mapping of the hospitals, clinics, and public health institutes that contributed to our collection of swab samples from influenza patients. Osaka area: Osaka Prefectural Institute of Public Health, National Hospital Organization Osaka National Hospital, Osaka City General Hospital, Higashiosaka City General Hospital, and Toyonaka Municipal Hospital. Tokyo area: National Center for Global Health and Medicine. Chiba area: Isumi Medical Center, Chiba Prefectural Togane Hospital, and its associated clinics. The distance between Osaka and Tokyo/Chiba is approximately 500 km, and an international airport is located in each of these areas.

Our sample collections were made in both the very early phase and the peak phase of the pandemic, as depicted in Figure 1A. Shortly after the first case of infection with the 2009 pdm influenza virus emerged in Japan, from May 16 to May 20, we collected 91 samples at the Osaka Prefectural Institute of Public Health (Figure 1B). By sequence analysis, we identified 46 of those samples as being positive for infection with the 2009 pdm influenza A(H1N1). These samples are referred to as samples “I” (Figure 1A).

About six months later, the influenza cases reported per sentinel peaked in the 48th epidemic week (Figure 1A). By this time, the 2009 pdm A(H1N1) viruses had crowded out other influenza viruses to become the dominant strains. From October 13, 2009, to January 6, 2010, we collected a total of 353 swab samples from patients with influenza-like illness at six different hospitals and eleven clinics in the Osaka, Tokyo, and Chiba areas (Figures 1A and 1B). Among the collected samples, 207 samples were detected as positive for infection with the 2009 pdm influenza A(H1N1) viruses and are referred to as samples “II.” Table 1 summarizes the 2009 pdm A(H1N1)-positive samples together with the names of the providing hospitals and clinics from where they were collected. Each sample was labeled with a code number specific for each of the hospitals and clinics listed in Table 1.

**Table 1:** Swab samples collected in this study.

Area	Hospital/Clinic (Code)	No. of samples	“I” or “II”	Sample collection period	Remarks
Osaka	Osaka Prefectural Institute of Public Health (OS)	46	“I”	May 16, 2009–May 20, 2009	
Osaka	National Hospital Organization Osaka National Hospital (OM)	16	“II”	Nov. 09, 2009–Dec 22, 2009	*1
Osaka	Osaka City General Hospital (OC)	21	“II”	Nov. 01, 2009–Dec. 01, 2009	
Osaka	Higashiosaka City General Hospital (HOH)	24	“II”	Oct. 27, 2009–Nov. 06, 2009	
Osaka	Toyonaka Municipal Hospital (TY)	18	“II”	Dec. 02, 2009–Jan. 04, 2010	
Tokyo	National Center for Global Health and Medicine (KK)	38	“II”	Oct. 20, 2009–Jan. 06, 2010	*2,*3
Chiba	Isumi Medical Center (IS)	20	“II”	Oct. 19, 2009–Nov. 24, 2009	
Chiba	Chiba Prefectural Togane Hospital (TO)	11	“II”	Oct. 14, 2009–Nov. 17, 2009	
Chiba	Takahashi Clinic (TA)	4	“II”	Oct. 20, 2009–Oct. 30, 2009	
Chiba	Sakuma Clinic (SA)	2	“II”	Oct. 20, 2009–Oct. 30, 2009	
Chiba	Okazaki Clinic (OK)	2	“II”	Oct. 20, 2009–Oct. 27, 2009	
Chiba	Matsuo Clinic (MA)	16	“II”	Oct. 13, 2009–Nov. 20, 2009	
Chiba	Ito Clinic (IT)	3	“II”	Oct. 13, 2009–Nov. 20, 2009	
Chiba	Furukawa Clinic (HU)	2	“II”	Oct. 22, 2009–Oct. 30, 2009	
Chiba	Hokushindo Sato Clinic (HO)	18	“II”	Oct. 20, 2009–Oct. 30, 2009	
Chiba	Hyuga Clinic (HI)	7	“II”	Oct. 13, 2009–Nov. 20, 2009	
Chiba	Haniya Clinic (HN)	4	“II”	Oct. 20, 2009–Oct. 30, 2009	
Chiba	Amano Clinic (AM)	1	“II”	Oct. 27, 2009	
	Total	253			

“I”, samples collected during May 16, 2009–May 20, 2009.

“II”, samples collected during October 13, 2009–January 06, 2010.

*1, N295S and A156T mutations in NA.

*2, H275Y mutation in NA (oseltamivir-resistance, severe case).

*3, D185N mutation in NA (fatal case).

Viral RNA was extracted from the samples, and multiple reverse-transcriptase PCRs were carried out. Partial sequences of the HA and NA segments were amplified by PCR and then sequenced (see more details in MATERIALS AND METHODS).

### Phylogenetic analysis and genetic characterization

**Figure 2:**
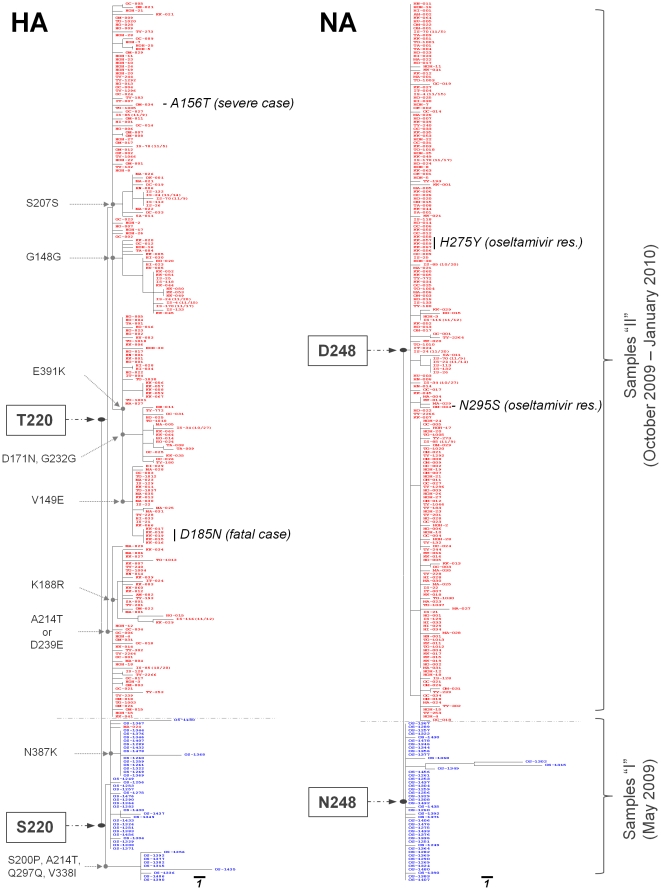
Maximum parsimony phylogenetic analysis with the partial sequences of HA and NA. Phylogenetic trees have been created for HA (left panel) and NA (right panel) proteins based on the partial sequences of the 2009 pdm A(H1N1) influenza viruses in a total of 253 samples that had been collected in May 2009 (samples “I”) and during the period of October 2009 to January 2010 (samples “II”). Samples “I” and “II” are marked in blue and red, respectively. For both HA and NA, the phylogenetic trees demonstrate two distinct clusters represented by samples “I” and “II”. The major non-synonymous or synonymous mutations are indicated. The scale bars represent 1 base substitution per site.

For a total of 253 samples, partial sequences of the HA and NA segments were analyzed by using the maximum parsimony method. As Figure 2 demonstrates, samples “I” and “II” were clearly distinguished by two clusters, where both HA S220T and NA N248D were identified as the major non-synonymous mutations that discriminate the samples between the two clusters. Other non-synonymous mutations were also discovered in samples “I” and “II” (Table 2), which define several sub-groups, especially in the HA segment (Figure 2).

**Table 2:** Amino acid changes observed in 2009 pdm A(H1N1) virus in samples of “I” and “II”.

Segment	Position	Samples “I” (number of samples)	Samples “II” (number of samples)
HA	145	S (46)	S (202), L (5)
HA	149	V (46)	V (183), E (24)
HA	156	A (46)	A (206), T (1)
HA	185	D (46)	D (202), N (5)
HA	188	K (46)	K (187), R (20)
HA	200	S (37), P (9)	S (207)
HA	203	A (46)	A (202), T (5)
HA	214	A (37), T (9)	A (158), T (48), V (1)
**HA**	**220** *	**S (46)**	**T (206), S (1)**
HA	222	R (46)	R (200), K (7)
HA	239	D (46)	D (204), E (3)
HA	250	L (46)	L (194), I (13)
HA	291	D (46)	D (190), N (17)
HA	321	P (46)	P (196), S (11)
HA	338	V (37), I (9)	V (206), I (1)
HA	387	N (32), K (13), R (1)	N (206), D (1)
HA	391	E (46)	E (145), K (61), G (1)
**NA**	**248** *	**N (46)**	**D (207)**
NA	275	H (46)	H (202)+Y (5)
NA	295	N (46)	N (206)+S (1)

Total of samples “I” = 46.

Total of samples “II” = 207.

*The positions of major amino acid substitutions that clearly discriminate between the 2009 pdm influenza viruses identified in samples “I” and those found in samples “II”.

**Figure 3:**
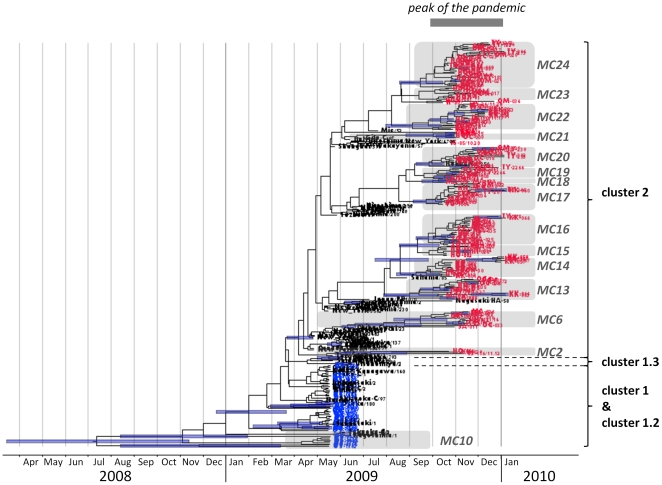
Bayesian MCMC inference with the concatenated HA and NA partial sequences of the 2009 pdm A(H1N1) influenza viruses. A Bayesian MCMC tree was computed with the concatenated partial sequences of HA and NA from the 2009 pdm A(H1N1) influenza viruses from Japan and the rest of the world for the period between May 2009 and January 2010. The clusters are named according to reference 17. The names of the samples collected in this study are marked in blue for samples “I” and in red for samples “II”. The names of the sequences retrieved from the NCBI Influenza Virus Resource database (60 from Japan and 18 from the rest of the world) are marked in black. The micro-clades (MC) we found in this study are highlighted in grey. The horizontal axis represents the time. The highest posterior density intervals (95%) of the node heights are marked by a light blue bar.

We conducted Bayesian coalescent Markov Chain Monte Carlo (MCMC) inference on the concatenated HA and NA partial sequences of the 253 samples we collected as well as the sequences retrieved from the NCBI Influenza Virus Resource database [18] (Figure 3; refer to Figure S1 for high resolution). We retrieved HA and NA sequences from samples collected in Japan and as well as worldwide from the NCBI Influenza Virus Resource database (Table S1). Both HA and NA sequences were available for a total of 58 Japanese isolates collected between May and September 2009, they are representing the four major clusters [17]. Additionally, we also retrieved the HA and NA sequences of two more Japanese isolates registered later in October 2009 as well as those of 18 worldwide members of cluster 2.

The mean evolutionary diversity values in the entire population for samples “I” (May 2009) and samples “II” (October 2009 to January 2010) as well as for a comparison with the Japanese isolates from a previous study (May to August, 2009) were 0.00346 (with a standard error estimate (S.E.) of 0.00058), 0.00359 (S.E. 0.00065), and 0.00243 (S.E. 0.00059), respectively. The evolutionary rate of the analyzed regions was calculated to be 8.09×10^−3^ substitutions per site per year. Tajima's D test for all Japanese samples has a value of −2.598 which indicates a purifying selection or a population-size expansion.

Although only a partial sequence of the influenza genome was analyzed in this study, our data of the Bayesian inference are essentially consistent with previously reported results from phylogenetic analyses [13], [14], [17]. In our study, however, clusters 1 and 1.2 are not distinguished, because the amino acid substitution NA-V106I that discriminates these two clusters (Table 3) was not involved in the sequence analysis of this study. Nevertheless, three micro-clades (MC) previously reported for the Japanese samples [17] are found in our phylogenetic tree; MC10 in cluster 1/1.2, and both MC2 and MC6 in cluster 2 (Figure 3). Samples “I” collected in the present study are grouped in the overlapping clusters 1/1.2, whereas all of the samples “II” are grouped in cluster 2. None of our samples were found in cluster 1.3. Some of the viruses in the samples “I” belong to MC10. In cluster 2, however, only a small number of viruses belong to MC2 and MC6. Moreover, it is important to note that the 2009 pdm A(H1N1) viruses continued to evolve further. Indeed, we could find at least 12 new micro-clades, named MC13 to MC24, as shown in Figure 3. Data with a higher resolution are available in Figure S1. Each micro-clade represents a group of viruses that share major common substitutions, as summarized in Table S2. Micro-clades found for our samples collected during the peak phase (October 2009–January 2010) greatly differed from those for the isolates collected from May 2009 until September 2009 in Japan (Table 4). These results strongly suggest that the 2009 pdm A(H1N1) viruses have evolved from cluster 2 and spread widely over the country during the peak period of the pandemic stage in Japan. We have further performed the analysis by using Maximum Likelihood phylogenetic inference to verify our findings (Figure S2). However, the micro-clades were not well disparate, as compared with the results of the Bayesian inference (Figure 3).

**Table 3:** Amino acid substitutions in HA and NA segments discriminating clusters.

	HA	NA	
	S220T	V106I	N248D
**cluster 1**	S	V	N
**cluster 1.2**	S	I	N
**cluster 1.3**	S	I	D
**cluster 2**	T	I	D

Data are from the present study and [13], [14].

**Table 4:** Comparison of the 2009 pdm A(H1N1) influenza viruses collected in Japan before and during the peak phase of the pandemic.

Cluster	Before peak phase (May–September 2009)	Peak phase (October 2009–January 2010)
1	**MC10 in samples “I”**, and [17]	-
1/1.2	**the other viruses (except MC10) in samples “I”**, and [17]	-
1.3	viruses [17]	-
2	MC2 [17]	**MC2 in samples “II”**
2	MC3 [17]	-
2	MC4 [17]	-
2	MC5 [17]	-
2	MC6 [17]	**MC6 in samples “II”**
2	MC7 [17]	-
2	MC8 [17]	-
2	MC9 [17]	-
2	Fukushima/1 [17]	-
2	Gifu-C/67 [17]	**MC21 in samples “II”**
2	Hiroshima/200 [17]	-
2	Hiroshima/230 [17]	-
2	Mie/41 [17]	-
2	Niigata/717 [17]	-
2	Niigata/749 [17]	-
2	Wakayama/57 [17]	-
2	-	Nagasaki/HA-58
2	-	**MC13 in samples “II”**
2	-	**MC14 in samples “II”**
2	-	**MC15 in samples “II”**
2	-	**MC16 in samples “II”**
2	-	**MC17 in samples “II”**
2	-	**MC18 in samples “II”**
2	-	**MC19 in samples “II”**
2	-	**MC20 in samples “II”**, Hokkaido/256
2	-	**MC22 in samples “II”**
2	-	**MC23 in samples “II”**
2	-	**MC24 in samples “II”**

Clusters and micro-clades (MC) 2 to 10 are named according to [17].

The sequences of Nagasaki/HA-58 and Hokkaido/256 that were not reported in [17] have been obtained from the Influenza Virus Resource database.

Synonymous and non-synonymous mutations in the partial sequences of HA and NA for the viruses presented in this table are summarized in Table S2.

### Comparison with the 1918 “Spanish flu” viruses

**Figure 4:**
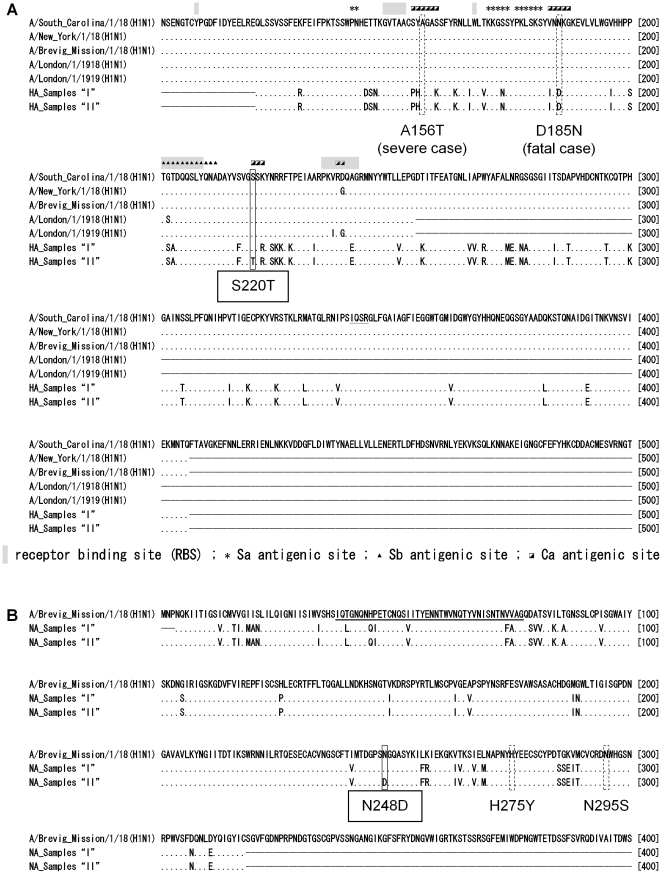
HA and NA sequence alignments between the 1918 H1N1 and 2009 pdm H1N1 viruses. (A) The HA alignment between the consensus sequences obtained for samples “I” and “II” and the five available sequences of 1918 H1N1 viruses. Also represented on this alignment are the major substitution (S220T) between samples “I” and “II”; A156T and D185N observed in fatal or severe cases; the antigenic sites Sa (*), Sb (▴), and Ca (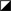); the receptor binding site (highlighted in grey); and the cleavage site (underlined). (B) The NA alignment between the consensus sequences obtained for samples “I” and “II” and that of the 1918 A/Brevig_Mission/1/18 (H1N1) virus. Also represented on this alignment are the major substitution (N248D) between samples “I” and “II,” H275Y and N295S causing oseltamivir resistance, and the stalk region (underlined).

In terms of the amino acid signatures reported by Pan *et al*. [11], the 2009 pdm influenza A(H1N1) virus exhibits a relatively high similarity with the 1918 “Spanish flu” viruses. Thus, we first examined the two major non-synonymous mutations, i.e., HA-S220T and NA-N248D. Interestingly, the 1918 “Spanish flu” viruses had HA (S220) and NA (N248) as did the 2009 pdm influenza A(H1N1) viruses in samples “I” (Figure 4). In contrast, HA (T220) and NA (D248) were not recorded in the 1918 “Spanish flu” viruses.

### Oseltamivir-resistance associated mutations: H275Y and N295S in NA

In this study, three patients were found to be infected with an oseltamivir-resistant influenza virus. The NA-H275Y mutation was found in two of these patients, whereas the NA-N295S mutation was found in one. All of these mutations were detected in samples “II”; none of them were in samples “I”. To our knowledge, the NA-N295S mutation is the first case so far reported for oseltamivir-resistance in the 2009 pdm influenza A(H1N1) viruses. The NA-N295S mutation was originally reported in drug-resistant H5N1 viruses [19]–[21]. In our clinical study carried out in Tokyo, the NA-H275Y mutation was found in a patient (6 years old) who had influenza-induced brain edema and severe pneumonia with little response to oseltamivir (4 mg/kg/day). The patient was subjected to steroid-pulse treatments in the ICU and was hospitalized for more than two months.

## Discussion

### Phylogenic analysis of 2009 pdm A(H1N1) viruses

The 2009 pdm influenza A(H1N1) virus was a new subtype of influenza virus that contained segments of genes from swine, avian, and human influenza viruses in a combination that has never been observed before in the world [8]. The HA gene of the 2009 pdm A(H1N1) viruses was reportedly derived from “classical swine H1N1” virus, which likely shares a common ancestor with the human H1N1 virus that caused the influenza pandemic in 1918, and whose descendant viruses continued to circulate in the human population with highly altered antigenicity of HA [7].

In this study, phylogenic analysis has revealed that HA-S220T and NA-N248D are the major non-synonymous mutations that clearly discriminate between the 2009 pdm influenza viruses identified in samples “I” and those found in samples “II” collected in Japan (Figure 2).

Studies on the worldwide evolution of the 2009 pdm A(H1N1) viruses have demonstrated four major clusters [13], [14], [17] (clusters classification and their respective amino acids substitutions are reported in Table S3). Likewise, the phylogenetic analysis carried out on Japanese isolates corroborates the circulation of those four clusters, whereas most of the Japanese isolates were further divided into 12 micro-clades [17]. To further study the circulation and evolution of these influenza viruses during the peak period in Japan, we investigated the sequences of 2009 pdm A(H1N1) influenza viruses that had been collected in various countries and registered in the NCBI Influenza Virus Resource database. In the present study, we found 14 micro-clades within the cluster 2 (Figure 3): 12 of them were new micro-clades, while two were previously reported.

The regions of the HA and NA segments used for the analyses in this study represented only about 10% of the whole influenza genome. We found an evolutionary rate for these regions of 8.09×10^−3^ substitutions per site per year. This value is even higher than the previously reported evolutionary rates for the 2009 pdm A(H1N1) (around 4.00×10^−3^ substitutions per site per year [15], [17]), which is in accordance with the higher variability of the HA and NA segments as compared with other segments in the whole genome of the 2009 pdm A(H1N1) viruses.

### Nonsynonymous mutations in HA

It is known that the H1 HA molecule has four distinct antigenic sites: Sa, Sb, Ca, and Cb [22], [23]. These sites of the human H1N1 viruses contain the most variable amino acids in the HA molecule, which have been subjected to antibody-mediated immune pressure since this virus was identified to have emerged in 1918 [7].

Figure 4A shows HA sequence alignments between the 1918 H1N1 viruses and 2009 pdm H1N1 viruses (samples “I” and “II”) obtained in this study. For this comparison, we refer to the HA sequences of the 1918 H1N1 pandemic strains that have been previously determined directly from autopsy materials of five infected people who died during the 1918–1919 pandemic [24]–[26]. As shown in Figure 4, several amino acid substitutions were observed in the antigenic sites of the HA protein. The above-mentioned mutation, HA-S220T, is located in the Ca antigenic site [23]. This nonsynonymous mutation was not observed in the 1918 H1N1 pandemic viruses.

Mutations of D239G/N (in H1 numbering) have reportedly been associated with severe cases [27]. Among the 253 samples we analyzed, however, none of these mutations were observed. Instead, a D239E mutation was found in three samples (Table 2). This mutation is reportedly not more virulent than the wild type [27].

Two severe clinical cases were reported in the National Hospital Organization Osaka National Hospital (Osaka) and the National Center for Global Health and Medicine (Tokyo). Sequence analyses of these two clinical isolates identified HA-A156T and HA-D185N, which are both located in the Ca antigenic site [23]. One of the above-mentioned cases was a fatal one, wherein a woman (72 years old) had preexisting autoimmune disease-associated liver cirrhosis. The amino acid residue 185 in the HA protein was asparagine (N), instead of aspartic acid (D), in both swab and tracheal fluid samples collected from the patient (Table S4). It is noteworthy that N is also the amino acid residue found in the HA protein of the reported sequences of the 1918 influenza viruses [24]–[26] (Table S4). Glycans located near antigenic peptide epitopes interfere with antibody recognition [28], and glycans near the proteolytic activation site of HA modulate cleavage and influence infectivity of the influenza virus [29]. Mutational deletion of HA glycosylation sites can affect viral receptor binding [30]. Therefore, it is of interest to examine whether *N*-linked glycosylation occurs at N185 in the HA protein and affects the infectivity of the influenza viruses.

### Oseltamivir-resistance associated mutations in NA

Studies with seasonal H1N1, H3N2, and the highly pathogenic avian H5N1 viruses revealed that single amino acid substitutions at several positions in or around the NA active site confer resistance to viruses against NA inhibitors [19]–[21], [31], [32]. Among these NA substitutions, a His-to-Tyr (H274Y) substitution at position 274 is one of the best characterized oseltamivir-resistance markers. The NA-H275Y substitution was detected in sporadic cases of oseltamivir-treated and –untreated patients infected with 2009 pdm A(H1N1) viruses [33]–[35]. It has recently been reported that the oseltamivir-resistant 2009 pdm influenza viruses were as pathogenic and transmittable as their drug-sensitive counterparts [36].

The widespread administration of oseltamivir would clearly contribute to the emergence of oseltamivir-resistant 2009 pdm influenza viruses as dominant variants. In this study, three patients were identified as being infected with oseltamivir-resistant mutant variants (two with NA-H275Y and one with NA-N295S) (Figure 2). The oseltamivir-resistance viruses were found in 1.2% of the total samples that were collected. Owing to heavy use of oseltamivir in Japan, however, this rate can be expected to greatly increase in the upcoming season, as exemplified by the emergence of oseltamivir-resistant influenza viruses in the previous seasons [37]–[39]. Therefore, it is important to continuously monitor for the emergence of oseltamivir-resistant 2009 pdm influenza A(H1N1) viruses.

### Structural insights into mutations of the HA and NA proteins

**Figure 5:**
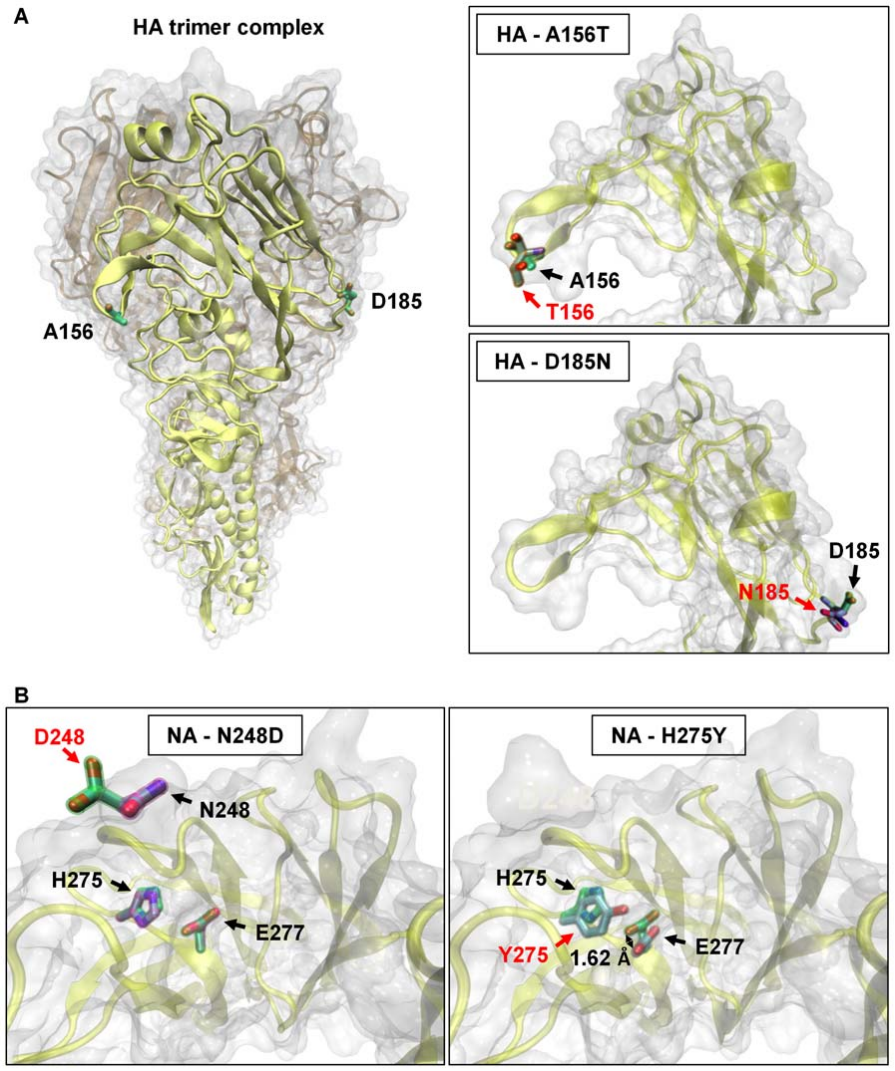
Structural modeling for HA and NA proteins with observed mutations. (A) The left panel depicts the three-dimensional structure of an HA trimer complex deduced from the crystal structure of the H1N1 hemagglutinin of the 2009 A(H1N1) virus (PDB entry: 3LZG) to highlight one HA monomer with the amino acid substitutions (A156T and D185N) found in our severe cases. The right panels represent close-up views of the head part of the HA monomer with an amino acid substitution of A156T (top) or D185N (bottom). Both A156T and D185N substitutions appear to occur in the Ca antigenic site and to head outside the receptor-binding site. (B) Two model structures of NA created from the crystal structure of N1 neuraminidase (PDB entry: 2HU4). The left panel shows the NA protein with the N248D substitution, representing locations of the amino acid substitution as well as H275 and E277. Substitution of N248 to D248 appears to have little effect on the side chain of E277. The right panel shows the NA protein with the H275Y substitution, revealing that the side chain of E277 is shifted by 1.62 Å in the oseltamivir-resistant variant found in samples “II.”

Crystal structures of the HA protein of the 2009 pdm A(H1N1) influenza virus are available [12], [40], and structural modeling of the NA protein has also been established on the basis of the crystal structure of the H5N1 virus [11]. Therefore, structural modeling analysis would facilitate our understanding of the possible effect of mutations in the 2009 pdm influenza viruses. Figure 5A depicts the model structure of HA protein, highlighting the amino acid substitutions at positions 156 and 185 that were found in severe cases, as described above. The models based on the crystal structure of H1 hemagglutinin from the 2009 pdm H1N1 virus (PDB entry: 3LZG) demonstrate that the amino acid substitutions of A156T and D185N are not located in the receptor-binding site, but rather in the Ca antigenic site (Figure 5A). It would be of interest, therefore, to examine whether vaccines produced by using the A/California/7/2009 (H1N1) virus with A156 and D185 in HA could exert their maximal effectiveness against those variants as well.

Pan *et al.* [11] have suggested that NA-N248D may be associated with oseltamivir resistance, since N248 is located closely to H275 (N1 numbering). The NA-H275Y mutation is known to cause oseltamivir resistance mainly because of its ability to reposition the carboxy side chains of E277, a critical residue for hydrogen bond formation with the drug molecule [21], [41]. Thus, we examined the potential effect of the NA-N248D (Figure 5B) by utilizing the crystal structure of the H5N1 neuraminidase (PDB entry: 2HU4) as a structural template. As compared with the notable effect of the NA-H275Y mutation, the substitution of N248 to D248 in our model caused little conformational change in the side chain of E277 (Figure 5B), which is consistent with hitherto available clinical data.

### Stalk motif in the NA protein

The importance of the NA stalk region in the replication of influenza viruses was pointed out by Castrucci *et al.* [42]. Recently, Zhou *et al.* have defined 6 stalk-motifs and suggested their potential role in the variation of virulence and pathogenicity of the avian H5N1 influenza [43]. Comparison of the stalk region motif of the 2009 pandemic influenza A(H1N1) viruses in samples “I” and “II” revealed its similarity to the “A/Gs/Gd/1/96/H5N1-like” motif as well as to the stalk motif of the 1918 H1N1 pandemic influenza virus (Figure S3). The “A/Gs/Gd/1/96/H5N1-like” motif was reported to be related to fast release but low pathogenicity of viruses in mice [43]. Likewise, similar results were reported for the 1918 pdm H1N1 viruses in poultry [44]. Although further studies are needed to prove the concept, these comparisons suggest that the 2009 pdm influenza A(H1N1) viruses resemble the 1918 pdm H1N1 and the H5N1 viruses.

### Conclusions

Hitherto, many studies have proven that the 2009 pdm influenza A(H1N1) viruses had a very fast mutation/evolution process with the emergence of numerous variants less than two months after the pandemic outbreak. In this study, sequence analysis has revealed that, among a variety of mutations, the HA-S220T and NA-N248D mutations are specific for the dominant variant(s) of the 2009 pdm influenza A(H1N1) viruses. Moreover, phylogenetic analysis of samples collected in Japan during the peak phase demonstrated the existence of 14 micro-clades, among which 12 were newly discovered in this study. The present study suggests that the 2009 pdm influenza A(H1N1) viruses have a genome with an extremely high evolutionary rate, and mutated viruses rapidly circulated around Japan via modern traffic networks.

## Materials and Methods

### Sample collection

Under written informed consent, we collected swab samples from patients with influenza-like symptoms at Chiba Prefectural Togane Hospital and its associated clinics, Isumi Medical Center, National Center for Global Health and Medicine, National Hospital Organization Osaka National Hospital, Toyonaka Municipal Hospital, Higashiosaka City General Hospital, Osaka City General Hospital, and Osaka Prefectural Institute of Public Health. Protocols for sample collection, storage and transportation to RIKEN needed for the present study were approved by the Institutional Review Boards at each hospital and organization. This clinical research was conducted according to the Declaration of Helsinki Principles. Sequence analysis for the viral RNA of influenza viruses obtained from swab samples was approved by the Research Ethical Committee at RIKEN Yokohama Institute.

### Sample preparation and sequencing

Viral RNA was extracted from samples with the QIAamp Viral RNA Mini Kit (QIAGEN K.K., Tokyo) according to the manufacturer's instructions. A multisegment Reverse Transcription-PCR step was then performed on extracted vRNA by using universal influenza A primers and with the conditions previously described by Zhou *et al.* [45] with the exception that we used 40 cycles, instead of 31, for the second cycle step. Amplifications by PCR of the regions of interest were performed with Takara Ex Taq (TaKaRa BIO INC, Kyoto) by using primers flanked with the T7 promoter for the forward primer and the SP6 promoter for the reverse primer. Two PCR primer sets were designed for the HA gene (positions 392 to 851 with 5′-taatacgactcactatagggGATTATGAGGAGCTAAGAGA-3′ and 5′-atttaggtgacactatagaaGATCCAGCATTTCTTTCCAT-3′, and positions 792 to 1251 with 5′-taatacgactcactatagggACTGGACACTAGTAGAGCCG-3′ and 5′-atttaggtgacactatagaaCTCTTTACCTACTGCTGTGA-3′) and one primer set for the neuraminidase gene (positions 417 to 976 with 5′- taatacgactcactatagggCCTTGGAATGCAGAACCTTC-3′ and 5′-atttaggtgacactatagaaGATTGTCTCCGAAAATCCCA-3′). Samples were then treated with ExoSAP-IT (GE Healthcare, Tokyo) and sequenced. To analyze the NA stalk region, the 3′ region in the NA segment was amplified by using PCR primers (positions 13 to 476 with 5′-taatacgactcactatagggACGCGTGATCAGCAAAAGCAGG-3′ and 5′-atttaggtgacactatagaaATTAGGGTTCGATATGGGCT-3′). The resulting PCR products were subjected to direct sequencing.

The names of isolates, collection dates, and accession numbers of retrieved sequences from the GenBank database are available in Table S1.

### Phylogenetic analyses

The sequences from HA (nucleotide 392 to 1251) and NA (nucleotide 417 to 976) were concatenated and aligned with ClustalW version 2.1 [46]. The mean nucleotide diversity was computed with MEGA 5.0 [47] by using the Maximum Composite Likelihood method. The standard error estimates were obtained with a bootstrap method of 1000 replicates. Tajima's D tests were performed with MEGA 5 [47]. For phylogenetic analysis, Bayesian Markov Chain Monte Carlo (MCMC), Maximum Likelihood, and Maximum Parsimony methods were used with two independent runs which were performed and compared to validate the resulting trees.

#### Bayesian Markov Chain Monte Carlo (MCMC)

The best-fit model of nucleotide substitution was estimated by using jModelTest 0.1.1 [48], [49]. BEAST package v1.6.1 [50] was used to perform the Bayesian MCMC inferred trees and calculate the evolutionary rate. The General Time-Reversible (GTR) model was used with a gamma parameter of 4 and invariant sites. A strict molecular clock with an exponential growth model was used. For each analysis, a chain length of 200,000,000 was used and sampled every 20,000 states. Convergence was confirmed with Tracer v1.5 [51]. The maximum clade credibility tree was annotated with TreeAnnotator (included in the BEAST package) with a 10% burn-in, and visualized with FigTree v1.3.1 [52].

#### Maximum Likelihood and Maximum Parsimony

MEGA 5.0 [47] was used to compute the Maximum Likelihood and Maximum Parsimony trees. Maximum Likelihood trees were computed with the general time reversible model (gamma distributed with invariant sites) and 1000 bootstrap replicates. A complete deletion of the gap and missing data information was applied.

### Analysis of model structures

Homology modeling of the HA and NA proteins was achieved with the Modeler 9v8 program [53] based on the previously reported crystal structures of HA (PDB entry: 3LZG) and NA (PDB entry: 2HU4). These crystal structures were chosen based on the amino acid sequence comparisons and the resolution of the structure. Model structures were aligned and analyzed by using the DeepView Swiss-PdbViewer v4.0.1 program [54], and structural pictures were created with the VMD 1.8.7 program [55].

## Supporting Information

Figure S1 **Bayesian MCMC tree.** Higher resolution of Figure 3.
(TIF)

Figure S2 **Maximum likelihood phylogenetic analysis with the concatenated HA and NA partial sequences of the 2009 pdm A(H1N1) influenza viruses.** The concatenated partial sequences of HA and NA from the 2009 pdm A(H1N1) influenza viruses from Japan for the period between May 2009 and January 2010 were used to compute the maximum likelihood tree. The clusters are represented in brackets, and the number of substitutions per site is indicated under the tree. The names of samples “I”, samples “II”, and sequences retrieved from the NCBI Influenza Virus Resource database are marked in blue, red, and black, respectively. Micro-clades previously reported by Shiino *et al.* (MC2, MC6, and MC10) [17] are highlighted in red. Bootstrap values for 1000 replicates are indicated and scale bar represents the number of base substitutions per site.
(TIF)

Figure S3 **NA stalk region comparison.** The NA stalk motif (amino acids 36 to 79) of the 2009 pdm A(H1N1) influenza viruses in samples “II” has been compared with the different motifs described by Zhou *et al.* [43]. A high homology was found with the A/Gs/Gd/1/96/H5N1-like motif. High similarity was also found with the stalk region in the 1918 “Spanish” pandemic influenza A virus (A/Brevig_Mission/1/18(H1N1). In the figure, red letters represent differences in the amino acid sequence of the NA stalk motif among the A/Gs/Gd/1/96/H5N1, 2009 pdm A(H1N1), and 1918 pdm A(H1N1) viruses.
(TIF)

Table S1 **The hitherto reported 2008 A(H1N1) viruses that were used for philogenetic analyses in this study.**
(XLS)

Table S2 **Synonymous and non-synonymous mutations in the HA and NA regions analyzed in this study.** Consensus sequence dereived from the first isolates collected in Japan (MC1) was used as a reference [17]. Major amino acid substitutions determining micro-clades, are highlighted in grey.
(XLS)

Table S3 **The hitherto reported mino acid substitutions that discrimate clusters for the 2009 pandemic influenza A(H1N1) viruses.** The nomenclature of the clusters follows the report of Shiino et al. [17]. Bold letters indicate the amino acid substitutions that were previously reported in the parcial sequences we analyzed in this study.
(XLS)

Table S4 **Fatal case D185N, comparison with 1918–1919 H1N1.**
(DOC)

## References

[1] Novel Swine-Origin Influenza A (H1N1) Virus Investigation Team (2009). Emergence of a novel swine-origin influenza A(H1N1) virus in humans.. N Engl J Med.

[2] Schnitzler SU, Schnitzler P (2009). An update on swine-origin influenza virus A/H1N1: a review.. Virus Genes.

[3] Centers for Disease Control and Prevention (CDC) (2009). Update: novel influenza A (H1N1) virus infections - worldwide, May 6, 2009.. MMWR Morb Mortal Wkly Rep.

[4] World Health Organization (WHO) (2010). Pandemic (H1N1) 2009 - update 97.. http://www.who.int/csr/don/2010_04_23a/en/index.html.

[5] World Health Organization (WHO) (2010). H1N1 in post-pandemic period.. http://www.who.int/mediacentre/news/statements/2010/h1n1_vpc_20100810/en/index.html.

[6] World Health Organization (WHO) (2010). Influenza - update 115.. http://www.who.int/csr/don/2010_08_27/en/index.html.

[7] Garten RJ, Davis CT, Russell CA, Shu B, Lindstrom S (2009). Antigenic and genetic characteristics of swine-origin 2009 A(H1N1) influenza viruses circulating in humans.. Science.

[8] Neumann G, Noda T, Kawaoka Y (2009). Emergence and pandemic potential of swine-origin H1N1 influenza virus.. Nature.

[9] Centers for Disease Control and Prevention (CDC) (2010). Update: influenza activity - United States, August 30, 2009–March 27, 2010, and composition of the 2010–11 influenza vaccine.. MMWR Morb Mortal Wkly Rep.

[10] Itoh Y, Shinya K, Kiso M, Watanabe T, Sakoda Y (2009). In vitro and in vivo characterization of new swine-origin H1N1 influenza viruses.. Nature.

[11] Pan C, Cheung B, Tan S, Li C, Li L (2010). Genomic signature and mutation trend analysis of pandemic (H1N1) 2009 influenza A virus.. PLoS One.

[12] Xu R, Ekiert DC, Krause JC, Hai R, Crowe JE (2010). Structural Basis of Preexisting Immunity to the 2009 H1N1 Pandemic Influenza Virus.. Science.

[13] Nelson M, Spiro D, Wentworth D, Beck E, Fan J (2009). The early diversification of influenza A/H1N1pdm.. PLoS Curr.

[14] Fereidouni SR, Beer M, Vahlenkamp T, Starick E (2009). Differentiation of two distinct clusters among currently circulating influenza A(H1N1) viruses, March–September 2009.. Euro Surveill.

[15] Smith GJ, Vijaykrishna D, Bahl J, Lycett SJ, Worobey M (2009). Origin and evolutionary genomics of the 2009 swine-origin H1N1 influenza A epidemic.. Nature.

[16] Infectious Disease Surveillance Center (IDSC) (2009). The novel influenza (in Japanese).. http://idsc.nih.go.jp/idwr/douko/2009d/18-19douko.html.

[17] Shiino T, Okabe N, Yasui Y, Sunagawa T, Ujike M (2010). Molecular evolutionary analysis of the influenza A(H1N1)pdm, May–September, 2009: temporal and spatial spreading profile of the viruses in Japan.. PLoS ONE.

[18] NCBI Influenza Virus Resource.. http://www.ncbi.nlm.nih.gov/genomes/FLU/FLU.html.

[19] Kiso M, Mitamura K, Sakai-Tagawa Y, Shiraishi K, Kawakami C (2004). Resistant influenza A viruses in children treated with oseltamivir: descriptive study.. Lancet.

[20] Le QM, Kiso M, Someya K, Sakai YT, Nguyen TH (2005). Avian flu: isolation of drug-resistant H5N1 virus.. Nature.

[21] Collins PJ, Haire LF, Lin YP, Liu J, Russell RJ (2008). Crystal structures of oseltamivir-resistant influenza virus neuraminidase mutants.. Nature.

[22] Sheerar MG, Easterday BC, Hinshaw VS (1989). Antigenic conservation of H1N1 swine influenza viruses.. J Gen Virol.

[23] Luoh SM, McGregor MW, Hinshaw VS (1992). Hemagglutinin mutations related to antigenic variation in H1 swine influenza viruses.. J Virol.

[24] Taubenberger JK, Reid AH, Krafft AE, Bijwaard KE, Fanning TG (1997). Initial genetic characterization of the 1918 “Spanish” influenza virus.. Science.

[25] Reid AH, Fanning TG, Hultin JV, Taubenberger JK (1999). Origin and evolution of the 1918 “Spanish” influenza virus hemagglutinin gene.. Proc Natl Acad Sci U S A.

[26] Reid AH, Janczewski TA, Lourens RM, Elliot AJ, Daniels RS (2003). 1918 influenza pandemic caused by highly conserved viruses with two receptor-binding variants.. Emerg Infect Dis.

[27] Kilander A, Rykkvin R, Dudman SG, Hungnes O (2010). Observed association between the HA1 mutation D222G in the 2009 pandemic influenza A(H1N1) virus and severe clinical outcome, Norway 2009–2010.. Euro Surveill.

[28] Skehel JJ, Stevens DJ, Daniels RS, Douglas AR, Knossow M (1984). A carbohydrate side-chain on hemagglutinins of Hong Kong influenza-viruses inhibits recognition by a monoclonal-antibody.. Proc Natl Acad Sci U S A.

[29] Deshpande KL, Fried VA, Ando M, Webster RG (1987). Glycosylation affects cleavage of an H5N2 influenza-virus hemagglutinin and regulates virulence.. Proc Natl Acad Sci U S A.

[30] Gunther I, Glatthaar B, Doller G, Garten W (1993). A H1-hemagglutinin of a human influenza A-virus with a carbohydrate-modulated receptor-binding site and an unusual cleavage site.. Virus Res.

[31] Gubareva LV, Kaiser L, Hayden FG (2000). Influenza virus neuraminidase inhibitors.. Lancet.

[32] Zambon M, Hayden FG (2001). Position statement: global neuraminidase inhibitor susceptibility network.. Antiviral Res.

[33] Centers for Disease Control and Prevention (CDC) (2009). Oseltamivir-resistant novel influenza A(H1N1) virus infection in two immunosuppressed patients – Seattle,Washington, 2009.. MMWR Morb Mortal Wkly Rep.

[34] Leung TW, Tai AL, Cheng PK, Kong MS, Lim W (2009). Detection of an oseltamivir-resistant pandemic influenza A/H1N1 virus in Hong Kong.. J Clin Virol.

[35] Le QM, Wertheim HF, Tran HD, van Doorn HR, Nguyen TH (2010). A community cluster of oseltamivir-resistant cases.. N Eng J Med.

[36] Kiso M, Shinya K, Shimojima M, Takano R, Takahashi K (2010). Characterization of oseltamivir-resistant 2009 H1N1 pandemic influenza A viruses.. PLoS Pathogens.

[37] World Health Organization (WHO) (2008). Influenza A(H1N1) virus resistance to oseltamivir - Last quarter 2007 to 17 April 2008.. http://www.who.int/csr/disease/influenza/H1N1ResistanceWeb20080417.pdf.

[38] Matsuzaki Y, Mizuta K, Aoki Y, Suto A, Abiko C (2010). A two-year survey of the oseltamivir-resistant influenza A(H1N1) virus in Yamagata, Japan and the clinical effectiveness of oseltamivir and zanamivir.. Virol J.

[39] Baranovich T, Saito R, Suzuki Y, Zaraket H, Dapat C (2010). Emergence of H274Y oseltamivir-resistant A(H1N1) influenza viruses in Japan during the 2008–2009 season.. J Clin Virol.

[40] Yang H, Carney P, Stevens J (2010). Structure and Receptor binding properties of a pandemic H1N1 virus hemagglutinin.. PLoS Curr Influenza.

[41] Das K, Aramini JM, Ma LC, Krug RM, Arnold E (2010). Structures of influenza A proteins and insights into antiviral drug targets.. Nat Struct Mol Biol.

[42] Castrucci MR, Kawaoka Y (1993). Biologic importance of neuraminidase stalk length in influenza A virus.. J Virol.

[43] Zhou H, Yu Z, Hu Y, Tu J, Zou W (2009). The special neuraminidase stalk-motif responsible for increased virulence and pathogenesis of H5N1 influenza A virus.. PLoS One.

[44] Munier S, Larcher T, Cormier-Aline F, Soubieux D, Su B (2010). A genetically engineered waterfowl influenza virus with a deletion in the stalk of the neuraminidase has increased virulence for chickens.. J Virol.

[45] Zhou B, Donnelly ME, Scholes DT, St George K, Hatta M (2009). Single-reaction genomic amplification accelerates sequencing and vaccine production for classical and swine origin human influenza A viruses.. J Virol.

[46] Larkin MA, Blackshields G, Brown NP, Chenna R, McGettigan PA (2007). Clustal W and Clustal X version 2.0.. Bioinformatics.

[47] Tamura K, Dudley J, Nei M, Kumar S (2007). MEGA4: Molecular Evolutionary Genetics Analysis (MEGA) software version 4.0.. Mol Biol Evol.

[48] Posada D (2008). jModelTest: phylogenetic model averaging.. Mol Biol Evol.

[49] Guindon S, Gascuel O (2003). A simple, fast, and accurate algorithm to estimate large phylogenies by maximum likelihood.. Syst Biol.

[50] Drummond AJ, Rambaut A (2007). BEAST: Bayesian evolutionary analysis by sampling trees.. BMC Evol Biol.

[51] Rambaut A, Drummond AJ (2007). http://beast.bio.ed.ac.uk/Tracer.

[52] Available: http://tree.bio.ed.ac.uk/software/figtree/

[53] Sali A, Potterton L, Yuan F, van Vlijmen H, Karplus M (1995). Evaluation of comparative protein modeling by MODELLER.. Proteins.

[54] Guex N, Peitsch MC (1997). SWISS-MODEL and the Swiss-PdbViewer: An environment for comparative protein modeling.. Electrophoresis.

[55] Humphrey W, Dalke A, Schulten K (1996). VMD: Visual Molecular Dynamics.. J Mol Graph.

