# Supplementary figures and images for: Mutation Analysis of 2009 Pandemic Influenza A(H1N1) Viruses Collected in Japan during the Peak Phase of the Pandemic
Source: PLoS One. 2011 Apr 29;6(4):e18956. doi: 10.1371/journal.pone.0018956 (PMC3084724; doi:10.1371/journal.pone.0018956)

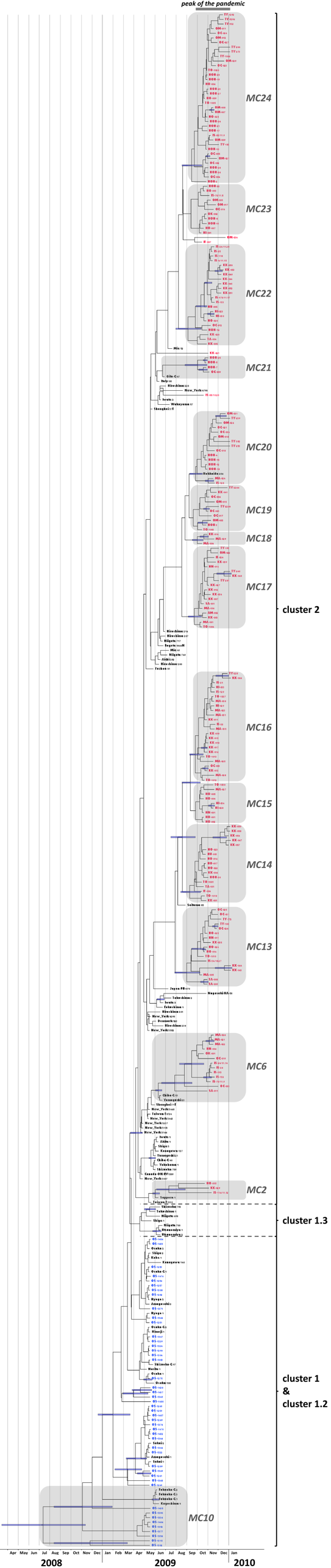

Supplement: Figure S1 — Bayesian MCMC tree. Higher resolution of Figure 3. (TIF) [file pone.0018956.s001.tif]

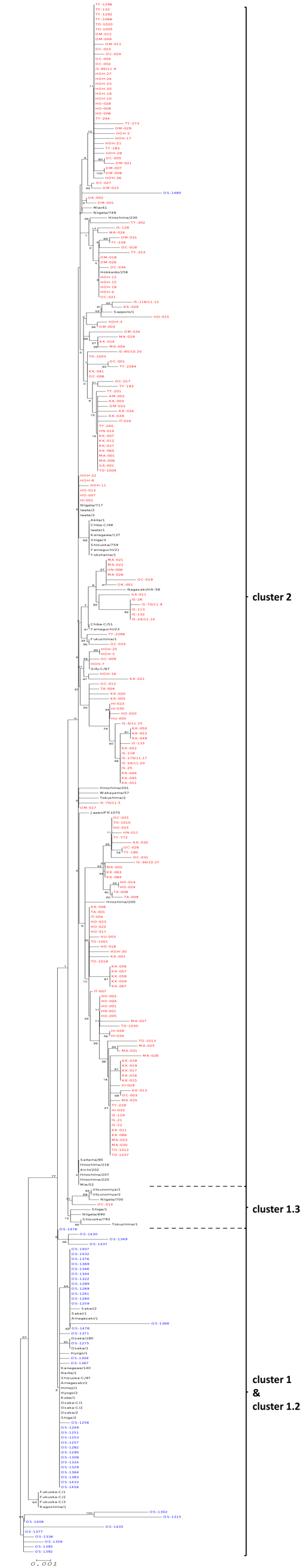

Supplement: Figure S2 — Maximum likelihood phylogenetic analysis with the concatenated HA and NA partial sequences of the 2009 pdm A(H1N1) influenza viruses. The concatenated partial sequences of HA and NA from the 2009 pdm A(H1N1) influenza viruses from Japan for the period between May 2009 and January 2010 were used to compute the maximum likelihood tree. The clusters are represented in brackets, and the number of substitutions per site is indicated under the tree. The names of samples “I”, samples “II”, and sequences retrieved from the NCBI Influenza Virus Resource database are marked in blue, red, and black, respectively. Micro-clades previously reported by Shiino et al. (MC2, MC6, and MC10) [17] are highlighted in red. Bootstrap values for 1000 replicates are indicated and scale bar represents the number of base substitutions per site. (TIF) [file pone.0018956.s002.tif]

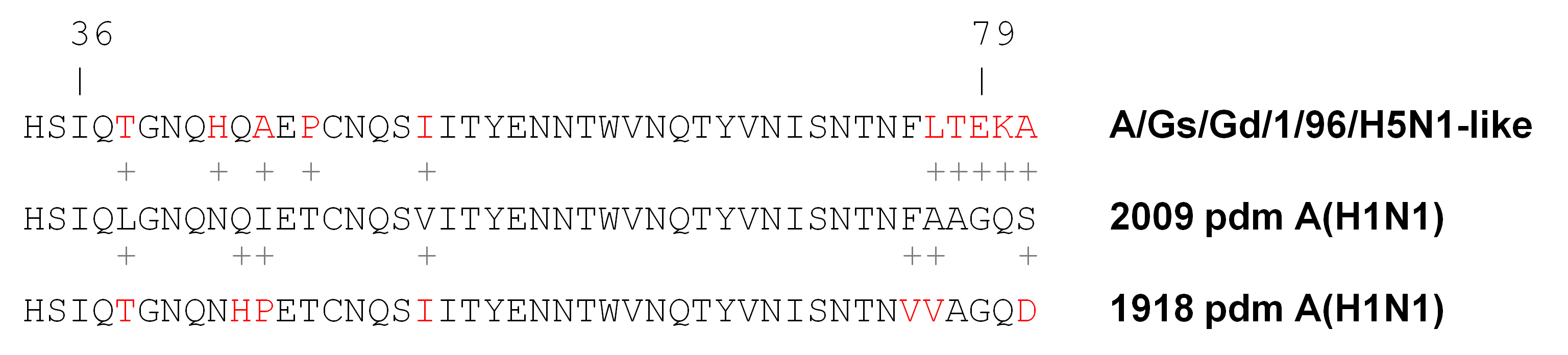

Supplement: Figure S3 — NA stalk region comparison. The NA stalk motif (amino acids 36 to 79) of the 2009 pdm A(H1N1) influenza viruses in samples “II” has been compared with the different motifs described by Zhou et al. [43]. A high homology was found with the A/Gs/Gd/1/96/H5N1-like motif. High similarity was also found with the stalk region in the 1918 “Spanish” pandemic influenza A virus (A/Brevig_Mission/1/18(H1N1). In the figure, red letters represent differences in the amino acid sequence of the NA stalk motif among the A/Gs/Gd/1/96/H5N1, 2009 pdm A(H1N1), and 1918 pdm A(H1N1) viruses. (TIF) [file pone.0018956.s003.tif]
