# Supplementary material for: Mutation Analysis of 2009 Pandemic Influenza A(H1N1) Viruses Collected in Japan during the Peak Phase of the Pandemic
Source: PLoS One. 2011 Apr 29;6(4):e18956. doi: 10.1371/journal.pone.0018956 (PMC3084724; doi:10.1371/journal.pone.0018956)
Supplement: Table S4 — Fatal case D185N, comparison with 1918–1919 H1N1. (DOC) [file pone.0018956.s007.doc]

**Table S4 - Fatal case D185N, comparison with 1918-1919 H1N1**

|  | Virus name | Amino Acid | Nucleic Acid |
| --- | --- | --- | --- |
| 1918-1919 | Influenza A virus (A/South Carolina/1/1918(H1N1)) | N | AAT |
|  | Influenza A virus (A/New_York/1/18(H1N1)) | N | AAT |
|  | Influenza A virus (A/Brevig Mission/1/1918(H1N1)) | N | AAT |
|  | Influenza A virus (A/London/1/1918(H1N1)) | N | AAT |
|  | Influenza A virus (A/London/1/1919(H1N1)) | N | AAT |
| 2009 | sample "I" | D | GAT |
|  | sample "II" | D | GAT |
|  | Fatal case | N | AAT |

Asn (N) encoded by AAT or AAC

Asp (D) encoded by GAT or GAC
